# Overreliance on EEG normalization: a case report of DEE-SWAS treated as presumed NCSE leading to avoidable escalation

**DOI:** 10.3389/fped.2026.1835013

**Published:** 2026-05-22

**Authors:** Jie Wu, Jie Deng, Manting Xu, Liyi Huang, Guangyuan Zhao

**Affiliations:** 1 Department of Emergency, Beijing Children’s Hospital, Capital Medical University, National Center for Children’s Health, Beijing, China; 2 Department of Neurology, Beijing Children’s Hospital, Capital Medical University, National Center for Children’s Health, Beijing, China

**Keywords:** anesthesia-induced coma, burst-suppression, DEE-SWAS, ESES, GRIA3

## Abstract

A 10-year-old boy with a 6-year history of recurrent seizures and cognitive decline was diagnosed with developmental and epileptic encephalopathy with spike-and-wave activation in sleep (DEE-SWAS). Previous treatments involving multiple anti-seizure medications and repeated high-dose methylprednisolone only provided temporary efficacy. Three weeks ago, he was misdiagnosed with non-convulsive status epilepticus (NCSE) at another hospital due to electrical status epilepticus during sleep (ESES) on electroencephalography (EEG). He was then put into an anesthesia-induced coma in order to convert the EEG to burst-suppression. However, this intervention resulted in severe complications, including ventilator-associated pneumonia. Discontinuing the anesthetics and sedatives, and adjusting his antiepileptic treatment regimens promoted his recovery. Although his seizures resolved, the ESES persisted on EEG. Furthermore, the prolonged induced coma impaired his neurological function, and the stay in intensive care unit imposed a significant medical burden. This case cautions carefully differentiating between ESES and NCSE, and against overreliance on EEG “normalization” without a rigorous risk-benefit assessment of anesthesia-induced coma.

## Introduction

Developmental and epileptic encephalopathy with spike-and-wave activation in sleep (DEE-SWAS) is a pediatric epilepsy syndrome with electrical status epilepticus during sleep (ESES) on electroencephalography (EEG). Its EEG abnormalities may not correlate with clinical seizures, so overreliance on EEG normalization could lead to overtreatment. Here, we report a case to highlight the importance of prioritizing clinical evaluation and to prompt reflection on the development of appropriate antiepileptic treatment regimens.

## Patient description

**Figure 1:**
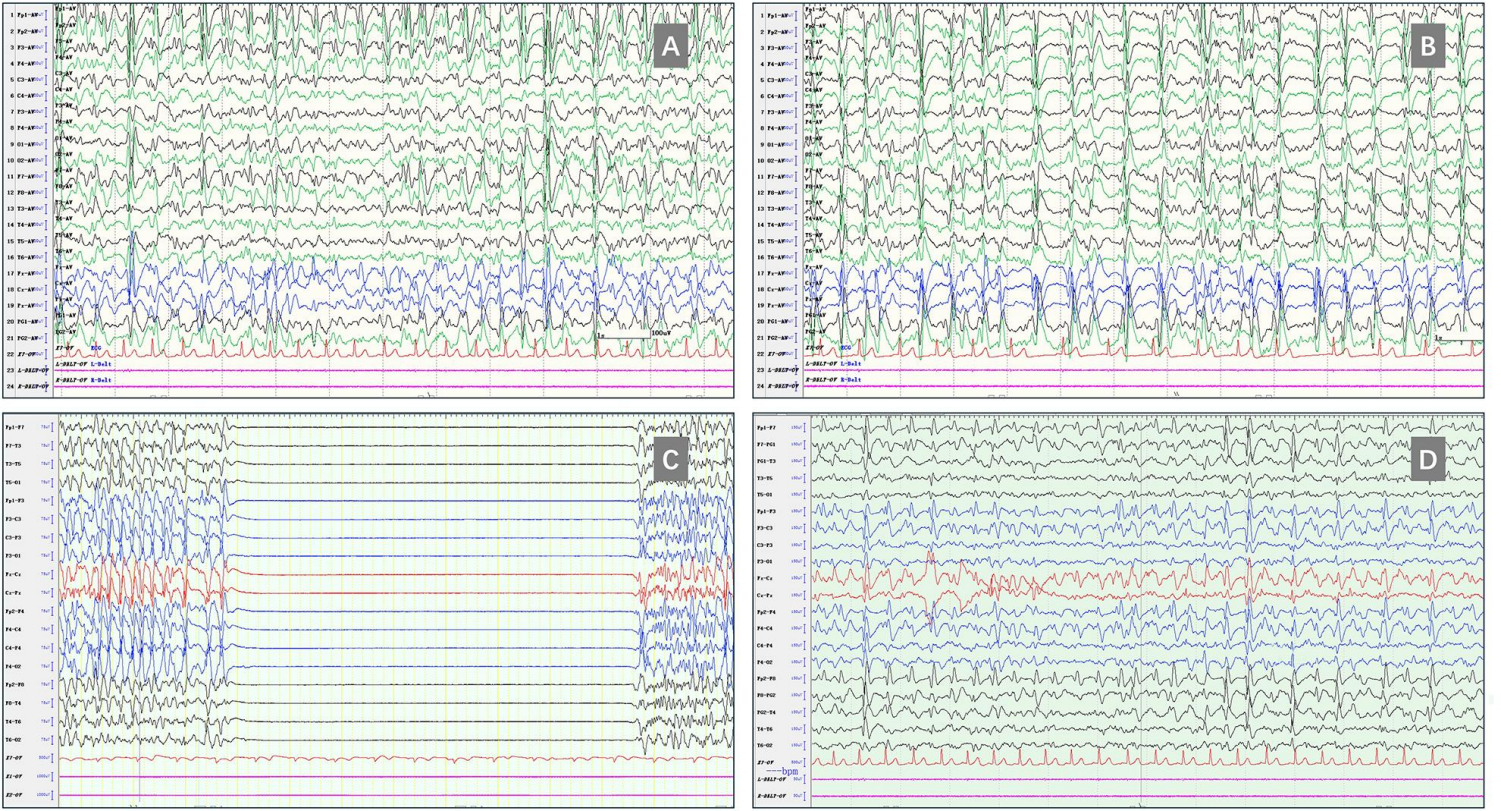
The EEGs of this patient before, during, and after the current hospitalization. **(A)** and **(B)** the interictal EEG recorded after the 10th high-dose corticosteroid treatment (May 2025, aged 10 years and 5 months, average montage, sens 20 *μ*V), **(A)** was recorded awake and **(B)** demonstrated continuous generalized spike-and-wave discharges during sleep. **(C)** The EEG recorded on the day of admission to our hospital showed burst suppression during the administration of midazolam, esketamine, and phenobarbital (3 July 2025, bipolar montage, 15 s/page, Sens 15 μV). **(D)** Pre-discharge EEG recheck revealed extensive interictal spike-and-wave discharges with no normal background activity (13 August 2025, bipolar montage, Sens 15 μV).

In July 2025, a 10-year-and-6-month-old boy was admitted to our hospital with recurrent seizures and cognitive regression for over 6 years. The patient's seizures began at the age of 4 years, including myoclonic seizures manifesting as quick hand shaking and negative myoclonic seizures as falls while walking, atypical absence seizures presenting as gaze and cessation of movements, and three episodes of focal to bilateral tonic-clonic seizures during sleep. Unsteady walking and slurred speech were also present, and 6 months later he was unable to stand, walk or speak. At the age of 5 years, the patient was admitted to the local hospital, where an EEG revealed frequent atypical absence seizures and a spike-wave index (SWI) of approximately 90% during the non-rapid eye movement (NREM) stage of sleep. This prompted the administration of intravenous methylprednisolone and sequential addition of sodium valproate (VPA), topiramate, clonazepam, and lamotrigine (LTG). After treatment, the boy experienced a seizure-free period up to 6 months. During that time, he was able to take care of himself, speak in full sentences, run and jump steadily. He started the first grade of elementary school although his academic performance was substandard, and he could also help his mother with simple farm work. However, at the age of 8 years, the patient's condition relapsed, with as many as twenty or more atypical absence seizures observed daily. Concurrently, he exhibited developmental regression, evidenced by a significant decline in verbal communication and ambulation. Repeated EEGs showed marked spike-and-wave discharges predominantly in the bilateral central and parietal regions, which generalized and increased during sleep, with SWI exceeding 85% and presenting as ESES. Therefore, he underwent ten courses of intravenous methylprednisolone (20 mg/kg/day for 3 consecutive days) from November 2023 to May 2025 at the local hospital, which achieved seizure reduction and restored his ability to walk. However, the efficacy lasted only 1 to 2 months with no significant EEG improvement (Figures 1A,B). Furthermore, he experienced adverse effects to corticosteroids, including weight gain and Cushing's syndrome, as well as fractures of the right radius and ulna. Before referral, the patient still had atypical absence seizures more than 10 times per day, manifesting as staring spells lasting several seconds (Supplementary Video 1). Between episodes, he could walk and speak single words but required assistance with feeding due to trembling hands.

Three weeks before being referred to our hospital, the patient was admitted to the neurology department of another general hospital in Beijing. An EEG showed widespread spike-and-wave discharges interictally, with SWI exceeding 90% during sleep. He was administered an intravenous injection of diazepam, followed by VPA and levetiracetam (LEV) infusions. His oral anti-seizure medications (ASMs) were adjusted to the following doses: VPA 22 mg/kg/day, LEV 22 mg/kg/day, LTG 2.2 mg/kg/day, and nitrazepam 0.2 mg/kg/day （body weight = 45 kg）. Intravenous phenobarbital proved ineffective in improving EEG, prompting the consideration of non-convulsive status epilepticus (NCSE). Consequently, he was intubated and placed on mechanical ventilation and subsequently transferred to the intensive care unit (ICU). A loading dose of midazolam (MDZ) was administered intravenously, followed by an infusion at a maintenance dose of 4.5 μg/kg/min, but there was still no change on the EEG. A further two loading doses of propofol were administered followed by continuous infusion, concurrently with bolus injections of phenobarbital every 8 h. Thereafter, the EEG transitioned to a burst-suppression pattern (Figure 1C). However, complications ensued after 150 h of burst-suppression maintenance, including hypotension, oliguria, and metabolic acidosis. This resulted in discontinuation of propofol and an increase in infusion dose of MDZ to 7 μg/kg/min, after which the EEG shifted to periodic discharges. Therefore, the patient was transferred by ambulance to our pediatric medical center with ventilator support, MDZ and methoxamine infusion, and admitted to the emergency ICU.

The boy is the only child of a healthy nonconsanguineous Chinese couple with an uneventful perinatal period. His motor development was normal but language development was mildly delayed. His mother's younger brother presented with seizures and developmental delay from early childhood and went missing at the age of 25 years; the mother's older brother and the maternal grandparents are healthy. Upon admission, physical examination revealed that the patient exhibited Cushing's appearance, with a Richmond Agitation-Sedation Scale score of −4. The pupillary light reflexes were slow bilaterally, the muscle strength and muscle tone of limbs were reduced, and the tendon reflexes were all weakened. Multiple multidrug-resistant bacteria were detected in bronchial lavage fluid cultures, including klebsiella pneumoniae, staphylococcus aureus and golden yellow bacillus. Pathogenic and autoimmune antibody testing of the cerebrospinal fluid were negative. Chest computed tomography scan revealed pneumonia and pleural effusion. Cranial magnetic resonance imaging showed mild cerebral atrophy. Whole-exome sequencing revealed that the patient carries a maternal variant of c.2337C > A/p.N779 K in the *GRIA3* gene. This variant is not present in the standard population database, and is not carried by his healthy maternal uncle or grandfather. According to the guidelines by the American College of Medical Genetics and Genomics (ACMG), the pathogenicity of this variant is interpreted as uncertain significance. The *GRIA3* encodes the A3 subunit of the glutamate *α*-amino-3-hydroxy-5-methyl-4-isoxazolepropionic acid (AMPA) receptor. It is associated with X-linked intellectual developmental disorder, WU-type, the phenotypes of which include seizures, intellectual disability, autism and interictal epileptic discharges on EEG.

The child was diagnosed with DEE-SWAS. As he was not experiencing clinical seizures, anesthesia was only administered to suppress the epileptiform discharges. However, the EEG deteriorated further and remained in the burst-suppression pattern after an intravenous bolus injection of MDZ (Supplementary Video 2). Furthermore, mechanical ventilation resulted in ventilator-associated pneumonia and multiple organ dysfunction including hyperammonemia, rhabdomyolysis, moderate anemia, liver dysfunction, hypoalbuminemia, and coagulation abnormalities. Therefore, the patient underwent three bronchoscopies and lavages and was treated with cefoperazone/sulbactam, vancomycin, tigecycline, polymyxin E, ceftazidime/avibactam, and aztreonam for infection. Due to two failed attempts at weaning from the ventilator caused by ICU acquired weakness, a tracheostomy was performed. Supportive therapy was provided, including an infusion of norepinephrine to maintain blood pressure, high-calorie milk via nasogastric feeding, and transfusions of red blood cell and albumin. Concurrently, phenobarbital was discontinued immediately, and the burst-suppression pattern disappeared the following day. MDZ and esketamine infusion doses were also gradually decreased. Given the suspection that the *GRIA3* variant may contribute to the pathogenesis, a strategy targeting glutamate receptors was attempted. Intravenous magnesium sulfate was administered to modulate glutamate receptors. Since LEV exhibits mild modulatory effects on AMPA receptors, its dosage was increased to 44.4 mg/kg/day. Meanwhile, the AMPA receptor antagonist perampanel was added and titrated rapidly to a maximum dose of 12 mg per night (0.27 mg/kg/day). For the other ASMs, VPA was maintained, LTG was tapered off, and clobazam was added to replace nitrazepam.

**Figure 2:**
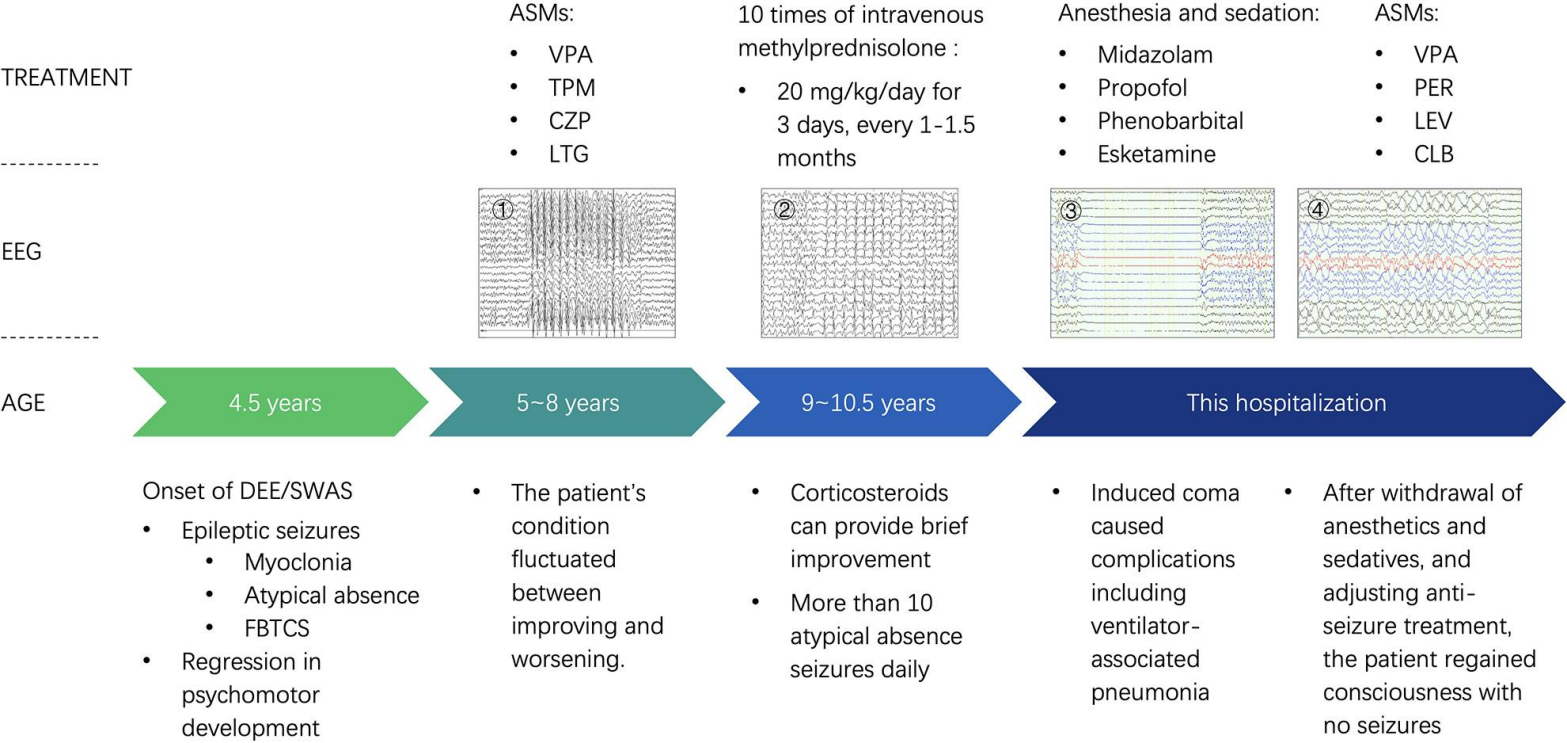
Timeline of the Patient's Course of Disease and Treatments. ① Ictal EEG recorded atypical absence seizure. ② Interictal EEG recorded after corticosteroid treatment showed generalized spike-and-wave discharges. ③ Under anesthesia and sedation, the EEG transitioned to burst-suppression. ④ After discontinuation of anesthesia and sedation, the burst-suppression resolved.

Following the aforementioned treatment, there was a gradual improvement in the patient's condition. On Day 18 of hospitalization, MDZ was completely tapered off. On Day 20, he regained consciousness and transitioned to artificial nasal breathing. Following his transfer to the neurology department, he demonstrated no further seizures and was able to respond with simple vocalizations and voluntary limb movements. On Day 30, he began taking semi-liquid food orally, and the tracheostomy tube was occluded. He was hospitalized for a total 43 days, was unable to stand and required a wheelchair at discharge (Figure 2). A rechecked EEG revealed findings consistent with those observed prior to treatment in Beijing, showing extensive interictal epileptiform discharges and ESES (Figure 1D). Presently, the boy has been seizure-free for 8 months. However, the previous tracheostomy has increased the burden of care on his mother, and he required assistance with feeding for 2 months following discharge. After consistent rehabilitation therapy, he is now able to walk independently and speak in simple sentences. His ASM regimen currently includes VPA, LEV, perampanel, and clobazam.

## Discussion

ESES refers to the increased frequency and generalization of interictal epileptiform discharges during non-rapid eye movement sleep. This EEG pattern is commonly observed in a group of epilepsy syndromes known as DEE-SWAS/epileptic encephalopathy (EE)-SWAS, which was diagnosed in this patient. These syndromes typically begin between toddlerhood and school age. Patients may experience multiple seizure types and combinations of cognitive, language, behavioral and motor regression. Clinical seizures and EEG abnormalities often resolve around puberty. However, the prognosis worsens with longer disease duration, especially if it exceeds two years (1). Due to their age-dependent nature, some non-pediatric neurologists may be unaware of these epilepsy syndromes. High-dose corticosteroids are used to treat ESES, but the effects are often difficult to sustain (2). Given their side effects, for example Cushing's syndrome and fractures experienced by this patient, careful attention should be paid to the dosage and course of the treatment. Furthermore, therapeutic approaches should focus on clinical symptoms, such as increased seizure frequency, multiple seizure types and cognitive impairment, rather than on EEG findings.

Some cases with DEE-SWAS/EE-SWAS have a genetic basis and may follow monogenic or complex inheritance. The major monogenic pathogenic gene is *GRIN2A*, which encodes the *α*2 subunit of the glutamate N-methyl-D-aspartic acid (NMDA) receptor (3). Therefore, a timely genetic diagnosis can facilitate the development of targeted therapeutic strategies. Ionotropic glutamate receptors are classified into three subfamilies, with their selective agonists being AMPA, NMDA and kainic acid, respectively (4). Subunit A3 of the glutamate AMPA receptor (AMPA-R) is encoded by the *GRIA3*, which is located at Xq25 (5). Rinaldi et al. evaluated the impact of forty-four *GRIA3* variants on AMPA-R function, revealing that gain-of-function variants are associated with more severe clinical phenotypes, including earlier onset of epilepsy, hypertonia and increased frequency of movement disorders (6). The pathogenicity of the maternal *GRIA3* variant carried by this patient is unclear, and functional verification is required to clarify it. However, his younger uncle's similar clinical history increases the likelihood that this variant contributes to the disease. As this is a missense variant, we considered the possibility of AMPA-R gain-of-function and adopted a therapeutic strategy to inhibit this receptor, which may have helped to control the patient's seizures. However, it is not possible to infer a causal relationship between genotype-informed therapy and improvement from this single case.

The treatment process for this patient at the other hospital warrants reflection. Without conclusive evidence of NCSE and to suppress EEG discharges alone, he underwent anesthesia-induced coma and prolonged maintenance of burst-suppression. This approach paradoxically necessitated mechanical ventilation, leading to secondary severe pulmonary infection and multiorgan dysfunction. Consequently, it exacerbated his neurological prognosis and imposed a significant burden on medical resources. The burst-suppression pattern refers to a continuous alternation between “burst phases” of high-voltage slow/sharp waves and “suppression phases” of depressed electrographic activity on EEG. It typically occurs in cortical injuries of various etiologies, correlates with severe impairment of consciousness, and represents a distinct state between continuous slow-wave sleep-like activity and an isoelectric EEG. Burst-suppression may lead to adverse consequences, including disruption of the blood-brain barrier, deepening of coma, and progression to irreversible neuronal damage (7, 8). For refractory and super-refractory status epilepticus, some guidelines recommend the induction of burst-suppression, evidence supporting this approach remains insufficient. Regarding the duration of therapeutic coma, the existing literatures have not reached a definitive conclusion. It is generally recommended to discontinue anesthetics 24–48 h after seizure control is achieved, as a shorter coma duration is relatively safer while reducing length of mechanical ventilation and ICU stay (9). This case reaffirms that EEG findings should not take precedence over clinical judgement, and that overreliance on EEG ‘normalization’ should be avoided without a rigorous risk-benefit assessment of anesthesia-induced coma.

## Conclusion

This DEE-SWAS case emphasises the importance of carefully differentiating between ESES and NCSE, and of avoiding EEG-driven treatment escalation in the absence of clear electroclinical evidence of NCSE. It also highlights the risks of overtreatment and advocates for balanced, personalized management to improve outcomes and reduce the medical burden.

## Data Availability

The datasets presented in this article are not readily available because of ethical and privacy restrictions. Requests to access the datasets should be directed to the corresponding author.
